# Long-Term Moderate Oxidative Stress Decreased Ovarian Reproductive Function by Reducing Follicle Quality and Progesterone Production

**DOI:** 10.1371/journal.pone.0162194

**Published:** 2016-09-27

**Authors:** Liangyan Shi, Jinjin Zhang, Zhiwen Lai, Yong Tian, Li Fang, Meng Wu, Jiaqiang Xiong, Xian Qin, Aiyue Luo, Shixuan Wang

**Affiliations:** 1 Department of Obstetrics and Gynecology, Tongji Hospital, Huazhong University of Science and Technology, Wuhan, Hubei, China; 2 Maternal and Child Health Hospital of Zigong, Sichuan, 643000, China; 3 The Central Hospital of Enshi Autonomous Prefecture, 158 Wuyang Road, Enshi Autonomous Prefecture, Hubei, 445000, China; Peking University Third Hospital, CHINA

## Abstract

Ovarian aging is a long-term and complex process associated with a decrease in follicular quantity and quality. The damaging effects of reactive oxygen species (ROS) in ovarian aging and ovarian aging-associated disorders have received relatively little attention. Thus, we assessed if the oxidative stress induced by long-term (defined by the Environmental Protection Agency as at least 30 days in duration) moderate ozone inhalation reduced ovarian reserves, decreased ovarian function and induced ovarian aging-associated disorders. The expression of oxidative stress markers and antioxidant enzymes was used to determine the degree of oxidative stress. Ultrastructural changes in ovarian cells were examined via electron microscopy. The ovarian reserve was assessed by measuring multiple parameters, such as the size of the primordial follicle pool and anti-Müllerian hormone (AMH) expression. The estrous cycle, hormone levels and fertility status were investigated to assess ovarian function. To investigate ovarian aging-associated disorders, we utilized bone density and cardiovascular ultrasonography in mice. The levels of oxidized metabolites, such as 8-hydroxy-2´-deoxyguanosine (8-OHdG), 4-hydroxynonenal (4-HNE) and nitrotyrosine (NTY), significantly increased in ovarian cells in response to increased oxidative stress. The ultrastructural analysis indicated that lipid droplet formation and the proportion of mitochondria with damaged membranes in granulosa cells were markedly increased in ozone-exposed mice when compared with the control group. Ozone exposure did not change the size of the primordial follicle pool or anti-Müllerian hormone (AMH) expression. The estrogen concentration remained normal; however, progesterone and testosterone levels decreased. The mice exposed to ozone inhalation exhibited a substantial decrease in fertility and fecundity. No differences were revealed by the bone density or cardiovascular ultrasounds. These findings suggest that the decreased female reproductive function caused by long-term moderate oxidative damage may be due to a decrease in follicle quality and progesterone production.

## Introduction

Female fertility declines with age. The gradual loss of fertility becomes more notable after age 35 and ends in menopause at a mean age of 50–51 years [1, 2]. The ovary exhibits an accelerated rate of aging compared with other body systems and demonstrates a gradual deterioration in the quantity and quality of its follicles [3].

In addition to a decrease in follicle number, a decrease in follicle quality may also cause reduced fertility. Several theories for understanding the cause of age-related changes have been developed. The concept of free radicals in aging, which was proposed by Denham Harman 50 years ago, has inspired a large amount of research [4]. The process of aging is characterized by a high concentration of endogenous reactive oxygen species and limited antioxidant activity that cause various oxidative injuries, such as lipid peroxidation of cell membranes, enzyme inactivation, protein oxidation, and DNA damage [5–11]. Evidence indicates that ROS and antioxidants disturb the normal physiological processes in the ovary; however, their importance in ovarian aging and ovarian aging-associated disorders has not been extensively investigated [12]. In women who received assisted reproductive treatment, the antioxidant activity in their oocytes, cumulus oophorus and follicular fluid decreased with age. Moreover, the concentration of ROS increased in these patients and was associated with worse outcomes [13–15]. These findings suggest that oxidative stress plays a role in the decrease in fertility with age.

According to the Environmental Protection Agency (EPA), ozone exposure for at least 30 days at ambient levels is defined as long-term exposure [16]. Previous studies suggested that an oxidative stress response can be induced by ozone inhalation in animal models [17, 18]. The increase in ROS caused by ozone may, in turn, activate a series of senescent phenotypes. In this study, we hypothesized that the oxidative stress induced by ozone inhalation is involved in ovarian aging; furthermore, we predicted that a single oxidative stress-inducing exposure would induce significant ovarian aging. Ovarian aging is a long and complex process associated with a decrease in follicular quantity and quality. Changes in hormone production caused by ovarian aging may produce various health consequences, including vasomotor symptoms, cardiovascular disease (CVD), osteoporosis, cognition, depression, mood disorders, sexual function, and vaginal atrophy [19]. To date, few studies have focused on the effects of oxidative stress on ovarian aging and ovarian aging-associated disorders in mice. Here, we discuss the result of ozone inhalation-induced oxidative stress with a focus on health outcomes, specifically general health conditions, ovarian aging, cardiovascular disease (CVD) and osteoporosis. The purpose of this report was to assess the effects of oxidative stress following long-term moderate ozone exposure on ovarian reserves, endocrine and reproductive function, and ovarian aging-associated disorders; in addition, we investigated the underlying mechanisms of these processes.

## Materials and Methods

### Ethics Statement

The experimental procedures were approved by the Animal Care Committee of Tongji Hospital within the Tongji Medical College at the Huazhong University of Science and Technology in China.

### Ozone Exposure

Six week old, specific pathogen-free, female C57BL/6J mice were obtained from Beijing HFK Bio-Technology Co., Ltd. (Beijing, PR China). The mice were maintained in a temperature-controlled environment, and rodent chow and water were provided *ab libitum*. Forty-eight mice were randomly divided into two groups: a normal control group (NC) and an ozone inhalation group (OI).

**Fig 1:**
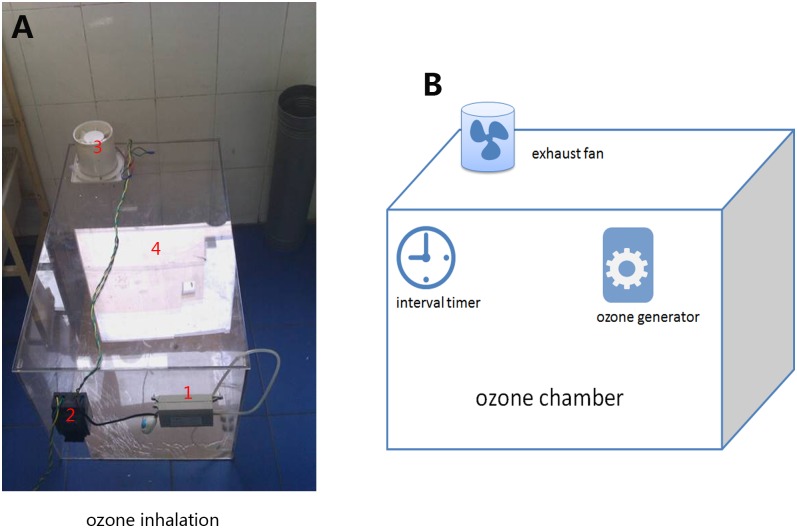
Picture and schematic representation of the ozone chamber and ozone generator. Note: 1. ozone generator; 2. interval timer; 3. exhaust fan; and 4. ozone chamber.

For the ozone inhalation paradigm, the mice were placed in a breeding box that contained an ozonator (Beijing Kang Er Xing Technology Development Co., Ltd., PR China). An ozone meter (Beijing Kang Er Xing Technology Development Co., Ltd., PR China) was used to measure the ozone concentration inside the cage during the experiment. A fan was used to maintain a constant ozone concentration of 1.2 mg/m^3^ via fresh airflow regulation. The mice in the OI group were exposed to ozone for 10 h (21:00–07:00), which was regulated by a time switch control, every day for 30 days [18, 20]. A diagram and picture of the ozone chamber and ozone generator are shown in Fig 1. Similar to the ozone inhalation group, mice from the NC group were placed in the same type of box, without the ozone generator, during the same time period every day for 30 days.

### Sample Collection

The mice were monitored daily during the entire treatment period. Once a week, the mice were weighed to calculate changes in body weight and observed to assess the condition of their fur. All mice were euthanized by CO2 inhalation on the first morning of metestrus after one month of treatment. The ovaries and blood samples were collected from twelve mice per group, and the ovaries were dissected and weighed for subsequent laboratory tests, such as western blotting, real-time polymerase chain reaction, and immunohistochemistry.

### Exhaustive Swimming Exercise

Six mice per group were subjected to an exhaustive swimming test. The mice were acclimated to the water environment and trained for swimming adaptation for five days prior to the experiment. Water was added to a plastic pool (93×58×58 cm) to a depth of 40 cm, and its temperature was maintained at 28±1°C. Endurance, defined as the time to reach exhaustion, was assessed by compelling the mice to swim in the plastic pool. Exhaustion was considered as the point when mice sank to the bottom of the pool without movement for 10 seconds and exhibited a lack of the righting reflex when they were placed on a flat surface [21, 22]. After the swimming test, the mice were euthanized, and the ovaries were dissected and fixed in 4% paraformaldehyde to determine the ovarian follicle counts.

### Immunohistochemistry

Five representative sections from each of the ten selected ovaries per group were used for immunohistochemistry to assess 8-hydroxy-2-deoxyguanosine (8-OHdG), 4-hydroxynonenal (4-HNE) and nitrotyrosine (NTY). The ovaries were fixed in 4% paraformaldehyde for 24 h, then subsequently placed in 70% ethanol and bathed in paraffin. The ovaries were consecutively cut into 5-μm thick slices, and every fifth section was transferred onto a slide. Immunohistochemistry of the ovary was performed using routine procedures as previously reported [23]. The primary antibodies were diluted to appropriate concentrations (8-OHdG, ab62623, 1:100 dilution, mouse monoclonal antibody, Abcam, Cambridge, MA, USA; 4-HNE, ab46545, 1:500 dilution, rabbit polyclonal antibody, Abcam, Cambridge, MA, USA; and NTY, ab7048, 1:50 dilution, rabbit polyclonal antibody, Millipore, Temecula, CA, USA) and incubated with the tissue sections at 4°C overnight. The sections were subsequently washed with PBS and incubated with secondary antibodies and a streptavidin-biotin peroxidase complex (SABC) (PK4001/PK4002, Zhongshan Golden Bridge Biotechnology, Zhongshan Golden Bridge, China). Negative controls were incubated with antibodies pre-absorbed with blocking peptide rather than the primary antibody. Due to age-related increases in oxidative damage [24], two-year-old mice were used as a positive control. Images were obtained using confocal microscopy (DM4000B; Leica, Germany).

### Western Blotting

The total expression of AMH and superoxide dismutase 2 (SOD2) was analyzed in the collected ovarian tissue. The proteins were extracted from ten ovaries per group. Tissue extracts (30 μg) were electrophoresed on a 10% SDS-PAGE gel and transferred to polyvinylidene difluoride membranes. The membranes were blocked using 5% nonfat milk in Tris-buffered saline (10 mM Tris and 150 mM phosphate buffered saline, pH 8.0) for 1 h at room temperature. Next, the membranes were incubated overnight at 4°C with diluted primary antibodies (AMH, AF1446, 1:250 dilution, goat antibody, R&D Systems, Minneapolis, USA; SOD2, ab68155, 1:1000 dilution, rabbit monoclonal antibody, Epitomics, California, USA; and actin, BM0005, 1:1000 dilution, rabbit polyclonal antibody, Boster, China). The blots were subsequently incubated with secondary antibodies (1:1000 dilution). The immunoreactive bands were observed using alkaline phosphatase (ALP) and BCIP/NBT staining. Each blot was completed in quadruplicate.

### Transmission Electron Microscopy

Immediately after dissection, five ovaries per group were fixed and weighed by immersion for 60 min in Karnovsky fixative (2.5% glutaraldehyde and 2% paraformaldehyde in 0.1 M Na-cacodylate buffer, pH 7.3) and prepared as previously described [25]. The ovaries were cut into approximately 1 mm^3^ pieces using a scalpel. After dehydration in an ascending ethanol series, the ovarian tissues were fixed in osmium tetroxide and embedded in epoxy resin. The sections were examined after Reynold’s lead citrate staining. Small follicles with visible oocyte nuclei were randomly selected, and images were obtained using a transmission electron microscope (CM 10 Philips, Eindhoven, The Netherlands) at 80 kV.

### RNA Isolation, Reverse Transcription and Real-Time Polymerase Chain Reaction

The ovaries were stored at −80°C. The RNA was randomly isolated from five ovaries per group to perform a real-time polymerase chain reaction (PCR) analysis. The total RNA of the ovaries was extracted with Trizol according to the manufacturer’s instructions (Invitrogen, Carlsbad, CA, USA). The RNA concentration was measured with a NanoDrop spectrophotometer (λ = 260/280 nm; ND 1000; NanoDrop Technologies, Wilmington, DE, USA). Real-time PCR was conducted using an AB StepOne Plus PCR machine (Applied Biosystems, Foster City, CA) as described in our previous study [23]. The relative gene amplification was determined with the 2^-ΔΔCT^ method [26]. The housekeeping gene glyceraldehyde-3-phosphate dehydrogenase (Gapdh) served as the endogenous control. The primer sequences for AMH were forward, 5'-TCCTACATCTGGCTGAAGTGATATG-3’ and reverse, 5'-CAGGTGGAGGCTCTTGGAACT-3'. The primer sequences for Gapdh were forward, 5'-TGTGTCCGTCGTGGATCTGA-3' and reverse, 5'-TTGCTGTTGAAGTCGCAGGAG-3'.

### Ovarian Follicle Counts

Ten paraffin-embedded ovaries per group were longitudinally and serially cut with a section thickness of 4 μm. Every fifth section was placed on a glass slide and stained with hematoxylin and eosin. Two individuals blinded to the treatment conditions analyzed the samples using a microscope. These two individuals have extensive knowledge of the morphology at each follicle stage. A histomorphometric assessment of follicle-containing oocytes was completed as previously described [27]. The experiment was repeated if the data from the two individuals varied widely. The data are represented as the primordial follicle number.

### Enzyme Immunoassays for 17β-Estradiol, Progesterone and Testosterone

The 17β-estradiol, progesterone and testosterone concentrations in mouse serum were determined using enzyme-linked immunoassay (EIA) kits (Cayman Chemical Company, Ann Arbor, USA) according to the manufacturer’s instructions and a spectrophotometer (Bio-Tek, Winooski, USA). For estrogen, the EIA typically exhibits an IC50 (50% B/B_0_) of approximately 125 pg/ml and a detection limit (80% B/B_0_) of approximately 20 pg/ml. For progesterone, the EIA typically exhibits an IC50 (50% B/B_0_) of approximately 70 pg/ml and a detection limit (80% B/B_0_) of approximately 10 pg/ml. The assay for testosterone has a detection range of 3.9–500 pg/ml and sensitivity (80% B/B_0_) of approximately 6 pg/ml. The within-group difference was 5.98%, whereas the between-group difference was 13.98%. These results indicate that this method has good specificity.

### Estrous Cycling and Fertility Status Tests

Six mice were used for estrous cycling and fertility status tests. The estrous cycle of individually housed mice was evaluated every morning for twenty days using vaginal cytology. Mating trials were initiated twenty days after the one-month treatment. These trials lasted for six months. The male mice were rotated at random among the cages during the mating trials. The male was removed from the cage as soon as a dam became pregnant. Following delivery, the dams were incorporated back into the mating trials. When the mating trials were complete, all mice were euthanized via CO_2_ inhalation.

### Bone Density Measurements

A bone mass analysis using mouse micro-CT was conducted with a SCANCO μCT50 (SCANCO Medical) on six mice per group following the fertility status tests. The scans were performed using the following instrument settings: E = 55 KVp, I = 110 μA, increment 7.4 μm, and threshold value = 375. Six mid-shaft tibia were used for the bone density analysis.

### Ultrasound Imaging and Doppler Echocardiography

Ultrasound scanning was conducted using a Vevo^®^1100 Imaging system on six mice per group. The mice were sedated with 1.5% isoflurane. While anesthetized, body temperature was maintained at 36–37°C, and the heart rate was 450–550 beats per minute (bpm). The B-mode and M-mode axial resolutions were 0.05–0.1 mm, and the B-mode lateral resolution was 0.2–0.5 mm (Zhou et al. 2002). The temporal frame rate in echo-mode was set to 60 Hz. A 1.0-mm sampling gate was used to obtain the inflow and outflow velocities, and the maximal sweep speed was 200 mm/sec. The spectral Doppler and M-mode images were recorded in multiple screen shots, and the Doppler and M-mode measurements were obtained from individual heart beats. The ultrasound parameters acquired from the spectral Doppler and the 2D and M-mode images are reported in the results section.

### Statistical Analysis

SPSS 17.0 software was used to conduct the statistical analyses. Differences in body weight, length of estrous cycles, exhausted swimming time, 17β-estradiol, progesterone and testosterone levels, follicle number counts, and bone density and cardiovascular ultrasound parameters were investigated using Student’s t-tests. Group differences in AMH and SOD2 expression determined using western blotting or PCR were analyzed using Student’s t-tests. Differences in the expression of 8-OHdG, 4-HNE and NTY between the experimental and control groups were examined using the non-parametric Kruskal–Wallis test. Values are represented as the mean ± SEM, and P<0.05 was considered statistically significant.

## Results

### Ozone Inhalation Stimulated Senescent Phenotypes in Mice

**Fig 2:**
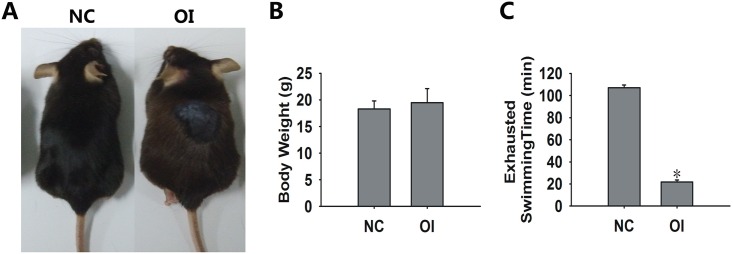
Body weight and exhausted swimming time for each group. (A) Representative images of mice in the NC and OI groups. (B) There was no significant difference in body weights between the two groups. (C) The exhausted swimming time of the OI mice was substantially decreased when compared with the control group (*P<0.05 when compared with the control group).

The mice were observed daily to closely monitor their health. All mice were alive at the end of the treatment, and no mice became severely ill. The physiological outcomes were analyzed and summarized. Compared with the NC group, the OI group exhibited aging phenomena, such as reduced exploratory activity and progressive greying of the hair and/or alopecia (Fig 2A). The weight of the OI group was not significantly different when compared with the NC group (Fig 2B). The latency to exhaustion during swimming was used an indicator of physical strength, and this parameter was substantially decreased in the OI group (Fig 2C).

### Ozone Inhalation Produced Oxidative Damage in the Ovary

**Fig 3:**
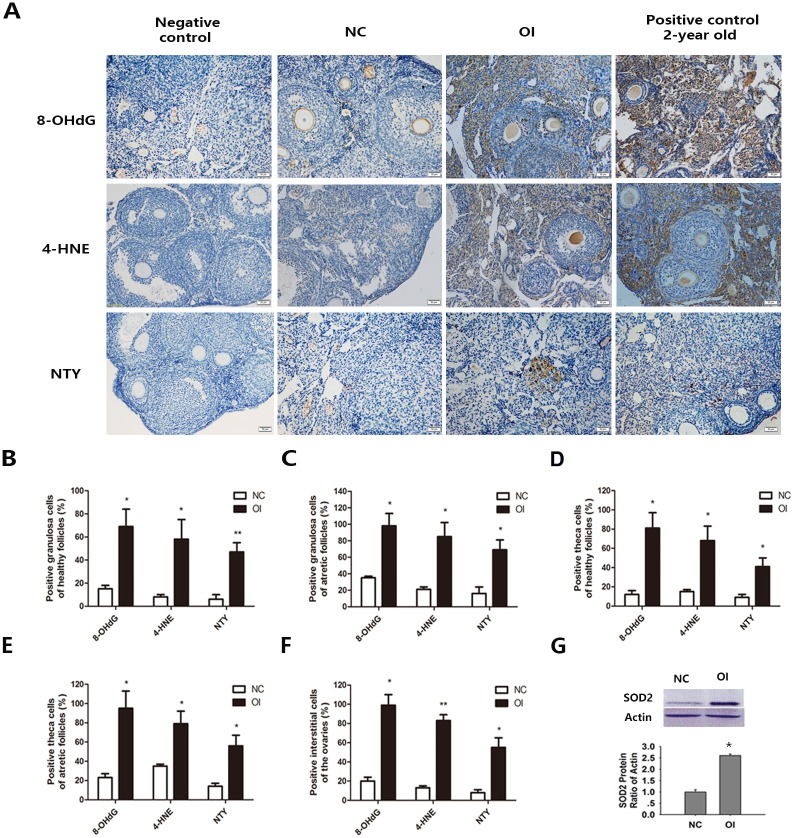
Effects of ozone inhalation on oxidative damage and antioxidant enzymes. (A) The immunostaining results (dark brown) for the oxidative damage in the interstitial cells and follicles in the ovaries of NC and OI mice, including oxidative DNA damage (8-OHdG), lipid peroxidation (4-HNE) and protein oxidation (NTY). (B-C) The percentage of positively-stained granulosa cells in healthy and atretic follicles. (D-E) The percentage of positively-stained theca cells in healthy and atretic follicles. (F) The percentage of positively-stained interstitial cells in the ovaries. (G) The protein expression of SOD2 was significantly increased following ozone exposure. Values represent the mean ± SEM, and the data were obtained from 3 independent experiments (*P<0.05 and **P<0.01 when compared with the NC group).

Oxidative lipid, protein, and DNA damage in interstitial cells and follicles of ozone-exposed mice were determined. 8-OHdG, one of the major products of DNA oxidation, is a widely accepted biomarker of oxidative DNA damage in biological systems and a potential marker of carcinogenesis. NTY, a product of tyrosine nitration, serves as an indicator of cell damage, inflammation and nitric oxide production. 4-HNE generated during the oxidation of lipids has been shown to modify mitochondrial proteins, which results in mitochondrial dysfunction. These molecules are widely accepted as biomarkers of oxidative DNA, protein, and lipid damage in biological systems [28]. Different follicular components and interstitial tissues were analyzed in the ovaries. Significant increases in 4-HNE, NTY, and 8-OHdG immunostaining in ovarian interstitial cells and all follicle components were observed in the OI group (Fig 3A). The percentage of positive staining for the indicated markers in granulosa cells/theca cells of healthy and atretic follicles was significantly increased when compared with the NC group (Fig 3B–3E). In addition, oxidative damage markers in interstitial cells were increased when compared with the NC group mice (Fig 3F). SOD2, one of the most important antioxidant enzymes in all tissues because of its defensive role against oxidative stress, was increased in the ovarian tissue of mice exposed to ozone inhalation (Fig 3G).

### Oxidative Stress Affected Follicle Ultrastructure in Mouse Ovaries

**Fig 4:**
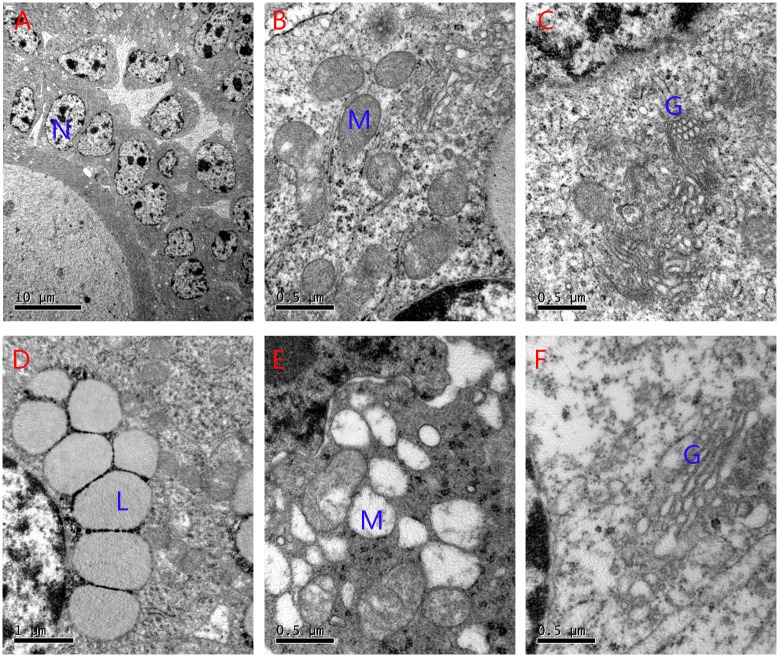
Ultrastructural changes in ovarian granulosa cells from both groups. (A-C), The normal mitochondria and Golgi complexes in the ovaries of NC mice. (D-F), The number of swollen mitochondria and Golgi complexes and the presence of lipid droplets increased in the OI group mice (N: nucleus of granulosa cells; M: mitochondrion; G: Golgi complex; and L: lipid).

To analyze the impact of oxidative stress on the organizational structure of cells, we assessed ultrastructural changes in the ovaries using electron microscopy (Fig 4A–4F). The mean areas of granulosa cells (GCs) and GC nuclei were not significantly different between groups (Table 1). However, there was a substantial difference in the morphology of mitochondria between groups. The cristae of the mitochondria were easily detected and appeared tubular in shape in the NC group, whereas most cells from the OI group showed highly electron-dense matrices and cristae that were barely visible. In the OI group, some mitochondria were swollen and showed vacuolization and degeneration of the cristae and matrix. Moreover, the OI group showed significant increases in swollen Golgi apparatus and lipid droplets.

**Table 1:** Ultrastructural morphology comparison of GCs from each group.

Variable	NC	OI
**Mean area of GCs (μm**^**2**^**)**	119.7±5.6	118.6 ±5.3
**Mean area of GC nuclei (μm**^**2**^**)**	23.6±2.3	22.4 ±1.5
**Mean nucleus/cell ratio**	0.21	0.22
**Cytoplasmic fraction of mitochondria (%)**	14.1±5.2	13.8±3.4
**Cytoplasmic fraction of lipid droplets (%)**	15.0±2.3	42±5.7**
**Cells with defective mitochondria**	4.7%	50.2%*

*P<0.05 when compared with the NC group.

**P<0.01 when compared with the NC group.

N = 5 per group. Values represent the mean±SD.

### Oxidative Stress Did Not Affect the Major Markers of Ovarian Reserves in Mice

**Fig 5:**
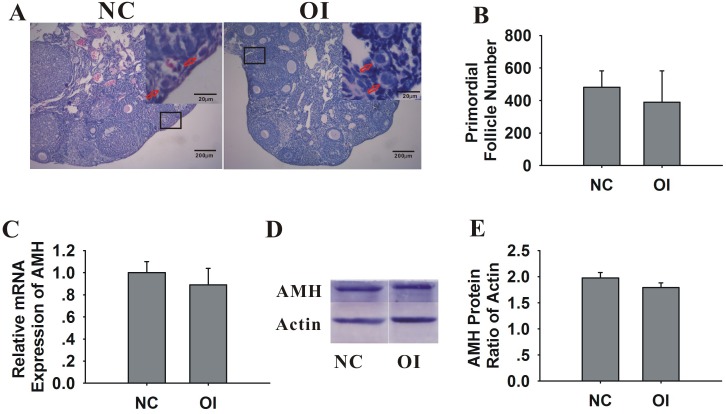
Effects of ozone inhalation on the ovarian reserves of mice. (A) Images of NC and OI mouse ovaries stained with hematoxylin and eosin. The inset (upper right corner of each group) indicates the number of primordial follicles (primordial follicles, red arrows). The images are shown at the original magnification of 100x. The insets are shown at a 400x magnification. (B) The bar graphs show similar numbers of primordial follicles in OI and NC mice. (C) The bar graphs show the mRNA expression of AMH in OI and NC mice. (D-E) The protein expression of AMH was determined using western blot analysis. The bar graphs show similar levels of AMH protein expression in the OI and NC mice. The data are expressed as the mean±SEM and were obtained from 3 independent experiments.

AMH serves as an ideal marker for ovarian reserves [29–33]. In addition to AMH, the counting of ovarian primordial follicles is another ideal parameter to evaluate the ovarian reserve [34, 35]. The hematoxylin and eosin-stained tissue (Fig 5A) revealed that the number of primordial follicles was similar between groups (Fig 5B). The AMH mRNA and protein expression in the ovaries was used to assess the ovarian reserve. No significant differences in the AMH mRNA or protein expression were identified between the OI and NC groups (Fig 5C–5E).

### Oxidative Stress Weakened the Ovarian Reproductive Function of Mice

Oxidative stress did not affect the ovarian reserves; thus, we assessed ovarian function by determining the estrous cycle length, serum estradiol and progesterone levels and reproductive capacity.

**Fig 6:**
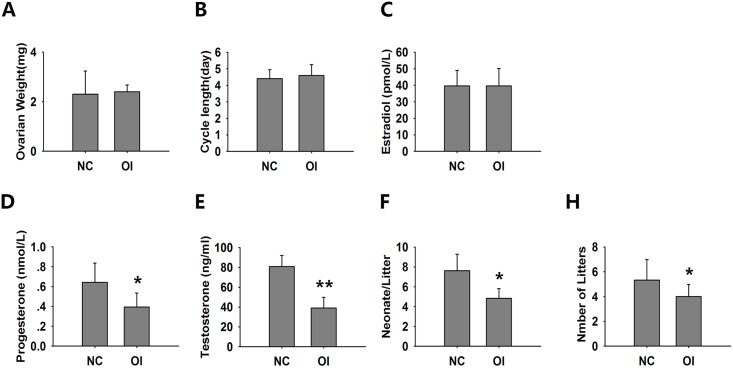
Assessment of ovarian, endocrine and reproductive function. (A) The ovarian weights of both groups. (B) The estrous cycles of the OI mice were normal when compared with the NC mice. Using EIA kits, the concentrations of 17β-estradiol (C) progesterone (D) and testosterone (E) were determined in duplicate. The data are shown as the mean ± SEM obtained from 6 animals. The mean litter size (F) and the mean number of litters (H) during a 6-month period were calculated as previously described. The OI mice showed decreased litter sizes and less overall litters when compared with the NC group. The data are shown as the mean ± SEM. N = 6 per group. * P<0.05 when compared with the NC.

The ovarian weight and estrous cycle of ozone-exposed mice were similar to NC mice (Fig 6A and 6B). The expression levels of the sex hormones estradiol and progesterone, which are secreted by granulosa and luteal cells, are typically used to evaluate ovarian endocrine function. Therefore, we determined the effect of ozone inhalation on the plasma levels of 17β-estradiol, progesterone and testosterone (Fig 6C–6E). The progesterone and testosterone levels were significantly decreased after ozone exposure; however, there was no significant difference in 17β-estradiol levels.

A decrease in fertility and fecundity is the most prominent marker of ovarian aging; thus, we analyzed the mean litter size and mean number of litters per group for 6 months after the treatment paradigm (Fig 6F and 6H). The fertility and fecundity was substantially decreased in the OI group.

### Oxidative Stress Had No Impact on Bone Density

**Fig 7:**
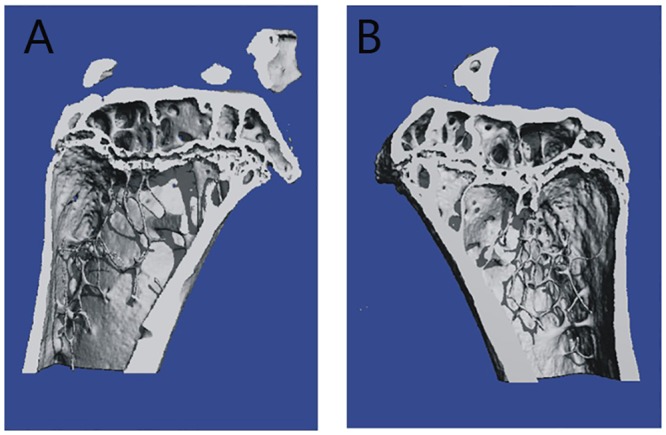
Bone mineral density (BMD) analysis of trabecular bone in the left tibia. (A) Representative micor-CT renderings show the trabecular bone of the NC group. (B) Representative micro-CT renderings show the trabecular bone of the OI group.

Osteoporosis, which is characterized by changes in the bone architecture, is the most common consequence of menopause. Therefore, we assessed the bone mass of mice from both groups 6 months after treatment. We used a SCANCO Micro-CT50 (SCANCO Medical) system to estimate the bone architecture via a trabecular bone analysis (Table 2). The trabecular bone volume (BV), connectivity density (ConnD) and trabeculae thickness (TbTh) in the tibia of the OI group were not significantly different (Fig 7B) when compared with the NC group (Fig 7A).

**Table 2:** Trabecular parameters of proximal tibia.

Variable	BV (mm^3^)	Tb.N. (1/mm)	Tb.Th. (mm)	Tb.Sp (mm)	ConnD (1/mm^3^)	SMI
**NC**	1.40±0.06	4.98±0.21	0.08±0.00	0.25±0.01	312.62±55.45	-2.62±0.25
**OI**	1.29±0.08	4.55±0.16	0.08±0.01	0.25±0.03	298.97±49.39	-1.65±1.15

The trabecular bone volume (BV), connectivity density (ConnD) and trabeculae thickness (TbTh) in the tibia of OI mice were not significantly different when compared with the NC group. N = 5 per group. Values represent the mean (±SD). BV, trabecular bone volume; TbN, number of trabeculae; TbTh, trabeculae thickness; TbSp, trabeculae separation; ConnD, connectivity density; and SMI, structure modeling index.

### Oxidative Stress Had No Effect on Cardiovascular Ultrasound Parameters

**Fig 8:**
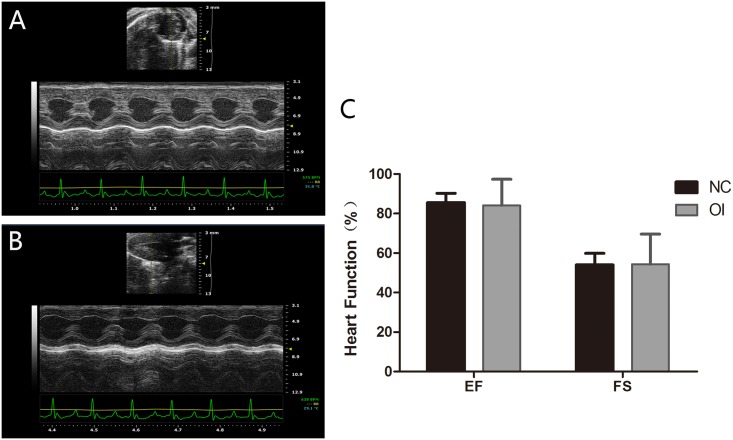
Ultrasound imaging used for the cardiovascular assessment of mice. (A) The cardiovascular ultrasound imaging of the NC group. (B) The cardiovascular ultrasound imaging of the OI group. (C) The cardiac function of the two groups was estimated by determining the EF and FS.

Cardiovascular disease is another common consequence of menopause. Thus, the ejection fraction (EF) and fractional shortening (FS) were examined to estimate the cardiac function of mice from both groups using a Vevo^®^1100 Imaging system. We found no significant changes despite the increased oxidative damage and decreased ovarian reproductive function (Fig 8A–8C).

## Discussion

ROS are an underlying factor of aging and may initiate aging by causing oxidative damage [36]. Studies in mice suggest that ozone exposure induces oxidative stress and results in many aging phenomena; thus, ozone exposure may be applicable in senescence research [17, 18, 37]. Our results demonstrated a significant increase in oxidative stress in mice after ozone inhalation using biochemical parameters. We observed signs of aging, including reduced exploratory activity and physical strength and a progressive greying of coat color. An exhaustive swimming exercise used to test physical strength revealed that physical strength was substantially decreased in the ozone-exposed mice. According to a study by Feng et al., ozone exposure decreases the physical strength of mice [18]. Thus, our results show the successful establishment of an oxidative stress model in mice via long-term ozone inhalation.

The ovarian aging process is marked by a gradual decrease in follicle quantity and quality. Increasing the level of endogenous ROS and decreasing antioxidant defenses leads to a wide range of cellular oxidative damage, including a subsequent decrease in ovarian quality. Jinhwan Lim’s study demonstrated that significant increases in oxidative damage to DNA, proteins and lipids in ovarian interstitial tissue could be used as markers of age-related changes [28]. Following ozone inhalation, the ovarian tissue of exposed mice showed increased expression of oxidative damage markers, including in 8-OHdG, 4-HNE and NTY. SOD2, an enzyme that converts superoxide anions to hydrogen peroxide, was markedly increased in the ozone-inhalation group when compared with the NC group. The alterations in these biomarkers confirm that ozone inhalation in mice increases oxidative damage to the ovaries.

Several clinical studies assessed the effect of follicular fluid on follicle quality and embryo formation; furthermore, ROS levels in follicular fluid were shown to be increased in unfertilized oocytes and poor quality embryos [14, 38]. In general, oxygen radicals, which increase with age, stimulate cellular damage. The mitochondria are the first organelles to degenerate because they are the site of oxygen radical production, and the accumulation of mitochondrial DNA deletions occurs with age [39, 40]. The substantially increased number of GCs with mitochondria degeneration and the loss of fertility in the OI group confirms that a decrease in follicle quality results from ROS-induced damage to follicular mitochondria [41].

The most notable characteristic of ovarian aging is the gradual loss of the primordial follicle pool [42]. However, we determined that the number of primordial follicles in the OI group of mice did not decrease after one month of ozone inhalation. The number of antral follicles decreases with age, and the serum levels of AMH become undetectable near menopause [43]. AMH is an excellent marker of ovarian reserves [29–33]. In our study, AMH expression was similar in the OI and NC groups. Our findings indicate that long-term moderate ozone inhalation had little effect on ovarian reserves, which may be due to enhanced antioxidant ability.

Menstrual changes are considered an early manifestation of ovarian dysfunction. During the menopausal transition, the menstrual cycle becomes increasingly irregular because there are insufficient FSH-sensitive follicles present in the ovaries [44, 45]. Rodents do not have menstrual cycles; however, they ovulate every estrous cycle when stimulated with FSH and transition through an age-related regression in their reproductive status. Previous work has demonstrated that important commonalities exist between primates and rodents in the hypothalamic control of ovarian function. The cycles of older mice become lengthened, and the capacity for cyclicity is subsequently lost. Most vaginal lavages from acyclic mice are leukocytic (i.e., diestrus or metestrus-2) [46]. In this study, all mice exhibited a 4–5 day estrous cycle, which was determined to be regular. Current evidence suggests that the estradiol level decreases comparatively late during ovarian aging, and the plasma concentration of progesterone does not significantly change [47–49]. Our results demonstrated that the plasma concentration of estradiol did not substantially change, whereas the progesterone decreased in OI mice. However, the fertility and fecundity significantly decreased in OI mice. Granulosa cells are the main source of estrogen and progesterone. Progesterone is also secreted by ovarian corpus luteum cells, which are luteinized granulosa cells [50, 51]. Oxidative stress plays a vital role in granulosa lutein insufficiency [52], which explains low progesterone levels in mice experiencing oxidative stress [53, 54]. An age-related decrease in egg quality is commonly considered the principal driving force for poor pregnancy success rates in aging females. Circulating progesterone is critical for embryo growth and the establishment and maintenance of pregnancy [55–57]. In our study, oxidative stress decreased the production of progesterone, which may comprise another important factor in decreased reproductive function. The levels of testosterone also decreased after ozone inhalation, which may be associated with decreased endocrine function of ovarian theca-interstitial cells in the ovary. However, the adrenal cortex is another major source of testosterone. Thus, the effects of adrenal cortex-derived androgen should be addressed.

Oxidative stress induced by ozone exposure may also exist in other body systems, including the brain, hypothalamus and pituitary; however, we did not determine the effects of ozone inhalation on the neuroendocrine axis. Changes in this axis may contribute to the ovarian aging observed in ozone exposed mice. Menopause may follow or precede age-related changes in the hypothalamus and central nervous system [58]. Oxidative stress to the ovary induced by ozone inhalation is the dominant phenomenon in our models; however, this is not a drawback because the models partially mimicked age-related physiological senescence in the body and successfully demonstrated the process of ovarian aging. To date, research on the causative factors of aging has mainly focused on the spontaneous damage accumulated during daily biological metabolism processes, which exist in nearly all organs [59]. Studies have shown that oxidative stress was triggered in mice exposed to ozone [17, 18]. Thus, we utilized ozone inhalation to accelerate the aging process. Moreover, the ovary, which was not the only tissue affected, undergoes substantially more serious aging effects when compared with other organs. In our experiment, the exposed dams exhibited normal estradiol levels and ovarian reserves, suggesting that a single induction of oxidative stress may not cause ovarian aging. Aging involves oxidative stress; however, not all oxidative stress may involve aging, especially ovarian aging.

Moreover, ovarian aging results in a decrease in gonadal steroids. These gonadal steroids are vital to maintain normal functioning of the reproductive tract and general female health, such as the maintenance of bone density, the cardiovascular system, cognition, wellbeing and sexuality. To investigate ovarian aging-associated disorders in mice, we examined bone density and cardiovascular function using ultrasound. We found no significant changes in the bone density or cardiovascular system of OI mice, which may be explained by the normal estradiol plasma levels.

In summary, our current findings suggest that the long-term moderate oxidative stress caused by ozone inhalation decreases the fertility and fecundity potential of mice. This effect may be due to poor follicle quality and decreased progesterone levels. Additional studies with large sample sizes of women with infertility are necessary to draw attention to the crucial role of oxidative stress in female reproduction. Notably, increasing the level of circulating progesterone may be a promising method to improve fertility and ART success.

## Supporting Information

S1 File Immunohistochemistry pictures for each marker. Significant increases in 4-HNE, NTY, and 8-OHdG immunostaining in ovarian interstitial cells and all follicle components were observed in the OI group.
(ZIP)

S2 File Transmission electron microscopy. The OI group showed significant increases in swollen mitochondria, Golgi apparatus and lipid droplets.
(ZIP)

S1 Table Bone density. The BV, ConnD and TbTh in the tibia of the OI group were not significantly different when compared with the NC group.
(XLSX)

S2 Table Concentration of estrogen. There was no significant difference in 17β-estradiol levels between the two groups.
(XLS)

S3 Table Concentration of progesterone. The progesterone levels were significantly decreased after ozone exposure.
(XLS)

S4 Table General condition. There was no significant difference in body weights between the two groups. The exhausted swimming time of the OI mice was substantially decreased when compared with the NC group.
(XLSX)

S5 Table Heart function. The cardiac function of the two groups was estimated by determining the EF and FS.
(XLSX)

S6 Table Litter experiment. The fertility and fecundity was substantially decreased in the OI group.
(XLSX)

S7 Table primordial follicle counts. The counting of ovarian primordial follicles showed no significant difference.
(XLSX)

## References

[1] Perheentupa A, Huhtaniemi I. Aging of the human ovary and testis. Molecular and cellular endocrinology. 2009;299(1):2–13. 10.1016/j.mce.2008.11.004 . 19059459

[2] Cetin MT, Kumtepe Y, Kiran H, Seydaoglu G. Factors affecting pregnancy in IVF: age and duration of embryo transfer. Reproductive biomedicine online. 2010;20(3):380–6. 10.1016/j.rbmo.2009.12.008 . 20117051

[3] Younis JS. Ovarian aging and implications for fertility female health. Minerva endocrinologica. 2012;37(1):41–57. . 22382614

[4] Harman D. Aging: a theory based on free radical and radiation chemistry. Journal of gerontology. 1956;11(3):298–300. . 13332224 10.1093/geronj/11.3.298

[5] Marchi S, Giorgi C, Suski JM, Agnoletto C, Bononi A, Bonora M, et al Mitochondria-ros crosstalk in the control of cell death and aging. Journal of signal transduction. 2012;2012:329635 10.1155/2012/329635 22175013 PMC3235816

[6] Brink TC, Demetrius L, Lehrach H, Adjaye J. Age-related transcriptional changes in gene expression in different organs of mice support the metabolic stability theory of aging. Biogerontology. 2009;10(5):549–64. 10.1007/s10522-008-9197-8 19031007 PMC2730443

[7] Cui H, Kong Y, Zhang H. Oxidative stress, mitochondrial dysfunction, and aging. Journal of signal transduction. 2012;2012:646354 10.1155/2012/646354 21977319 PMC3184498

[8] Ischiropoulos H. Free radical-induced damage to DNA: mechanisms and measurement. Free Radic Biol Med. 2002 6;32(11):1102–15. 12031895 10.1016/s0891-5849(02)00826-2

[9] Ischiropoulos H. Biological selectivity and functional aspects of protein tyrosine nitration. Biochem Biophys Res Commun. 2003 6;305(3):776–83. 12763060 10.1016/s0006-291x(03)00814-3

[10] H B. Effect of diet on cancer development: is oxidative DNA damage a biomarker? Free Radic Biol Med. 2002 5 15;32(10):968–74. 12008112 10.1016/s0891-5849(02)00808-0

[11] D MJ. The oxidative environment and protein damage. Biochimica et biophysica acta. 2005 1 17;1703(2):93–109. 15680218 10.1016/j.bbapap.2004.08.007

[12] Guérin P EM S, Ménézo Y. Oxidative stress and protection against reactive oxygen species in the pre-implantation embryo and its surroundings. Hum Reprod Update. 2001 Mar-Apr;7(2):175–89. 11284661 10.1093/humupd/7.2.175

[13] Hammadeh ME, Al Hasani S, Rosenbaum P, Schmidt W, Fischer Hammadeh C. Reactive oxygen species, total antioxidant concentration of seminal plasma and their effect on sperm parameters and outcome of IVF/ICSI patients. Archives of gynecology and obstetrics. 2008;277(6):515–26. 10.1007/s00404-007-0507-1 . 18026972

[14] Das S, Chattopadhyay R, Ghosh S, Ghosh S, Goswami SK, Chakravarty BN, et al Reactive oxygen species level in follicular fluid—embryo quality marker in IVF? Hum Reprod. 2006;21(9):2403–7. 10.1093/humrep/del156 . 16861701

[15] Oyawoye O, Abdel Gadir A, Garner A, Constantinovici N, Perrett C, Hardiman P. Antioxidants and reactive oxygen species in follicular fluid of women undergoing IVF: relationship to outcome. Human reproduction. 2003;18(11):2270–4. . 14585872 10.1093/humrep/deg450

[16] Prueitt RL L H, Zu K, Sax SN, Venditti FJ, Goodman JE. Weight-of-evidence evaluation of long-term ozone exposure and cardiovascular effects. Crit Rev Toxicol. 2014 10;44(9):791–822. 10.3109/10408444.2014.937855 25257962

[17] Kadiiska MB, Basu S, Brot N, Cooper C, Saari Csallany A, Davies MJ, et al Biomarkers of oxidative stress study V: ozone exposure of rats and its effect on lipids, proteins, and DNA in plasma and urine. Free radical biology & medicine. 2013;61:408–15. 10.1016/j.freeradbiomed.2013.04.023 23608465 PMC3968235

[18] Feng R, He W, Ochi H. A new murine oxidative stress model associated with senescence. Mechanisms of ageing and development. 2001;122(6):547–59. . 11295171 10.1016/s0047-6374(01)00232-9

[19] Stevenson JC. A woman's journey through the reproductive, transitional and postmenopausal periods of life: impact on cardiovascular and musculo-skeletal risk and the role of estrogen replacement. Maturitas. 2011;70(2):197–205. 10.1016/j.maturitas.2011.05.017 . 21788109

[20] Rivas-Arancibia S, Guevara-Guzman R, Lopez-Vidal Y, Rodriguez-Martinez E, Zanardo-Gomes M, Angoa-Perez M, et al Oxidative stress caused by ozone exposure induces loss of brain repair in the hippocampus of adult rats. Toxicological sciences: an official journal of the Society of Toxicology. 2010;113(1):187–97. 10.1093/toxsci/kfp252 . 19833740

[21] Zhang XL R F, Huang W, Ding RT, Zhou QS, Liu XW. Anti-fatigue activity of extracts of stem bark from Acanthopanax senticosus. Molecules. 2010 12;16(1):28–37. 10.3390/molecules16010028 21187815 PMC6259449

[22] Wu JL W Q, Huang JM, Chen R, Cai M, Tan JB. Effects of L-malate on physical stamina and activities of enzymes related to the malate-aspartate shuttle in liver of mice. Physiol Res. 2007;56(2):213–20. 16555951 10.33549/physiolres.930937

[23] Yang S, Wang S, Luo A, Ding T, Lai Z, Shen W, et al Expression patterns and regulatory functions of microRNAs during the initiation of primordial follicle development in the neonatal mouse ovary. Biol Reprod. 2013;89(5):126 Epub 2013/08/30. biolreprod.113.107730 [pii] 10.1095/biolreprod.113.107730 . 23986572

[24] Luderer U La J. Oxidative Damage Increases and Antioxidant Gene Expression Decreases with Aging in the Mouse Ovary. Biology of reproduction. 2011 4;84(4):775–82. 10.1095/biolreprod.110.088583 21148108 PMC3062040

[25] Makabe S, Naguro T, Stallone T. Oocyte-follicle cell interactions during ovarian follicle development, as seen by high resolution scanning and transmission electron microscopy in humans. Microsc Res Tech. 2006;69(6):436–49. Epub 2006/05/24. 10.1002/jemt.20303 . 16718658

[26] Aerts JL, Christiaens MR, Vandekerckhove P. Evaluation of progesterone receptor expression in eosinophils using real-time quantitative PCR. Biochim Biophys Acta. 2002;1571(3):167–72. Epub 2002/07/02. S0304416502001927 [pii]. . 12090930 10.1016/s0304-4165(02)00192-7

[27] Tilly JL. Ovarian follicle counts—not as simple as 1, 2, 3. Reproductive biology and endocrinology: RB&E. 2003;1:11 12646064 10.1186/1477-7827-1-11 PMC151785

[28] Lim J, Luderer U. Oxidative damage increases and antioxidant gene expression decreases with aging in the mouse ovary. Biology of reproduction. 2011;84(4):775–82. 10.1095/biolreprod.110.088583 21148108 PMC3062040

[29] Steiner AZ, editor Biomarkers of Ovarian Reserve as Predictors of Reproductive Potential. Seminars in reproductive medicine; 2013: Thieme Medical Publishers. 10.1055/s-0033-1356479 24101224

[30] Broekmans FJ K J, Hendriks DJ, Mol BW, Lambalk CB. A systematic review of tests predicting ovarian reserve and IVF outcome. Hum Reprod Update. 2006 Nov-Dec;12(6):685–718. 16891297 10.1093/humupd/dml034

[31] Fanchin R S L, Righini C, Guibourdenche J, Frydman R, Taieb J. Serum anti-Müllerian hormone is more strongly related to ovarian follicular status than serum inhibin B, estradiol, FSH and LH on day 3. Hum Reprod. 2003 2;18(2):323–7. 12571168 10.1093/humrep/deg042

[32] van Rooij IA B F, te Velde ER, Fauser BC, Bancsi LF, de Jong FH, Themmen AP. Serum anti-Mullerian hormone levels: a novel measure of ovarian reserve. Hum Reprod. 2002;17(12):3065–71. 12456604 10.1093/humrep/17.12.3065

[33] Koskela S T J. Anti-Müllerian hormone—a marker of ovarian function. Duodecim. 2016;132(3):226–32. 26951026

[34] Broekmans F J K EA, te Velde E R, et al Female reproductive ageing: Current knowledge and future trends. Trends Endocrinol Metab. 2007, 18: 58–65. 17275321 10.1016/j.tem.2007.01.004

[35] Djahanbakhch O E M, Zosmer A. Reproductive ageing in women. J Pathol. 2007, 211: 219–231. 17200943 10.1002/path.2108

[36] Harman D. The free radical theory of aging. Antioxidants & redox signaling. 2003;5(5):557–61. 10.1089/152308603770310202 . 14580310

[37] Alexeeff SE, Litonjua AA, Wright RO, Baccarelli A, Suh H, Sparrow D, et al Ozone exposure, antioxidant genes, and lung function in an elderly cohort: VA normative aging study. Occupational and environmental medicine. 2008;65(11):736–42. 10.1136/oem.2007.035253 18524839 PMC2575239

[38] Agarwal A, Gupta S, Sharma R. Oxidative stress and its implications in female infertility—a clinician's perspective. Reproductive biomedicine online. 2005;11(5):641–50. . 16409717 10.1016/s1472-6483(10)61174-1

[39] Kitagawa T, Suganuma N, Nawa A, Kikkawa F, Tanaka M, Ozawa T, et al Rapid accumulation of deleted mitochondrial deoxyribonucleic acid in postmenopausal ovaries. Biology of reproduction. 1993;49(4):730–6. . 8218635 10.1095/biolreprod49.4.730

[40] Keefe DL, Niven-Fairchild T, Powell S, Buradagunta S. Mitochondrial deoxyribonucleic acid deletions in oocytes and reproductive aging in women. Fertility and sterility. 1995;64(3):577–83. . 7641914

[41] Chattopadhayay R, Ganesh A, Samanta J, Jana SK, Chakravarty BN, Chaudhury K. Effect of follicular fluid oxidative stress on meiotic spindle formation in infertile women with polycystic ovarian syndrome. Gynecologic and obstetric investigation. 2010;69(3):197–202. 10.1159/000270900 . 20051691

[42] Coxworth JE, Hawkes K. Ovarian follicle loss in humans and mice: lessons from statistical model comparison. Human reproduction. 2010;25(7):1796–805. 10.1093/humrep/deq136 . 20504871

[43] Freeman EW, Sammel MD, Lin H, Gracia CR. Anti-mullerian hormone as a predictor of time to menopause in late reproductive age women. The Journal of clinical endocrinology and metabolism. 2012;97(5):1673–80. 10.1210/jc.2011-3032 22378815 PMC3339896

[44] Warburton D. Biological aging and the etiology of aneuploidy. Cytogenetic and genome research. 2005;111(3–4):266–72. 10.1159/000086899 . 16192704

[45] Soules MR, Sherman S, Parrott E, Rebar R, Santoro N, Utian W, et al Executive summary: Stages of Reproductive Aging Workshop (STRAW). Climacteric: the journal of the International Menopause Society. 2001;4(4):267–72. . 11770182

[46] Nelson JF, Gosden RG, Felicio LS. Effect of dietary restriction on estrous cyclicity and follicular reserves in aging C57BL/6J mice. Biology of reproduction. 1985;32(3):515–22. . 4039610 10.1095/biolreprod32.3.515

[47] Mersereau JE, Evans ML, Moore DH, Liu JH, Thomas MA, Rebar RW, et al Luteal phase estrogen is decreased in regularly menstruating older women compared with a reference population of younger women. Menopause. 2008;15(3):482–6. 10.1097/gme.0b013e31815982cf . 18202592

[48] Lee SJ, Lenton EA, Sexton L, Cooke ID. The effect of age on the cyclical patterns of plasma LH, FSH, oestradiol and progesterone in women with regular menstrual cycles. Human reproduction. 1988;3(7):851–5. . 3141454 10.1093/oxfordjournals.humrep.a136796

[49] Muttukrishna S, Child T, Lockwood GM, Groome NP, Barlow DH, Ledger WL. Serum concentrations of dimeric inhibins, activin A, gonadotrophins and ovarian steroids during the menstrual cycle in older women. Human reproduction. 2000;15(3):549–56. . 10686195 10.1093/humrep/15.3.549

[50] Denkova R B V, Staneva-Dobrovski L, Zvetkova E, Baleva K, Yaneva E, Nikolov B, Ivanov I, Simeonov K, Timeva T, Yankov M. In vitro effects of inhibin on apoptosis and apoptosis related proteins in human ovarian granulosa cells. Endocr Regul. 2004 6;38(2):51–5. 15497928

[51] Matsuda F I N, Manabe N, Ohkura S. Follicular growth and atresia in mammalian ovaries: regulation by survival and death of granulosa cells. J Reprod Dev. 2012;58(1):44–50. 22450284 10.1262/jrd.2011-012

[52] Lund SA M J, Van Kirk EA, Murdoch WJ. Mitogenic and antioxidant mechanisms of estradiol action in preovulatory ovine follicles: relevance to luteal function. Biology of reproduction. 1999 8;61(2):388–92. 10411516 10.1095/biolreprod61.2.388

[53] Robeck TR G C, Doescher BM, Sweeney J, De Laender P, Van Elk CE, O'Brien JK. Altrenogest and progesterone therapy during pregnancy in bottlenose dolphins (Tursiops truncatus) with progesterone insufficiency. J Zoo Wildl Med. 2012 6;43(2):296–308. 22779233 10.1638/2011-0166.1

[54] Günzel-Apel A U C, Wolf K, Einspanier A, Oei C, Piechotta M. Serum progesterone in pregnant bitches supplemented with progestin because of expected or suspected luteal insufficiency. Reprod Domest Anim. 2012 12;47(Suppl 6):55–60. 10.1111/rda.12029 23279466

[55] Stephens SB, Tolson KP, Rouse ML Jr., Poling MC, Hashimoto-Partyka MK, Mellon PL, et al Absent Progesterone Signaling in Kisspeptin Neurons Disrupts the LH Surge and Impairs Fertility in Female Mice. Endocrinology. 2015;156(9):3091–7. 10.1210/en.2015-1300 26076042 PMC4541622

[56] Wiltbank MC, Souza AH, Carvalho PD, Cunha AP, Giordano JO, Fricke PM, et al Physiological and practical effects of progesterone on reproduction in dairy cattle. Animal: an international journal of animal bioscience. 2014;8 Suppl 1:70–81. 10.1017/S1751731114000585 . 24703103

[57] Mesen TB, Young SL. Progesterone and the luteal phase: a requisite to reproduction. Obstetrics and gynecology clinics of North America. 2015;42(1):135–51. 10.1016/j.ogc.2014.10.003 25681845 PMC4436586

[58] Kermath BA, Gore AC. Neuroendocrine control of the transition to reproductive senescence: lessons learned from the female rodent model. Neuroendocrinology. 2012;96(1):1–12. 10.1159/000335994 000335994. 22354218 PMC3574559

[59] Sohal RS. Role of oxidative stress and protein oxidation in the aging process. Free radical biology & medicine. 2002;33(1):37–44. . 12086680 10.1016/s0891-5849(02)00856-0

