# Supplementary figures and images for: Long-Term Moderate Oxidative Stress Decreased Ovarian Reproductive Function by Reducing Follicle Quality and Progesterone Production
Source: PLoS One. 2016 Sep 27;11(9):e0162194. doi: 10.1371/journal.pone.0162194 (PMC5038974; doi:10.1371/journal.pone.0162194)

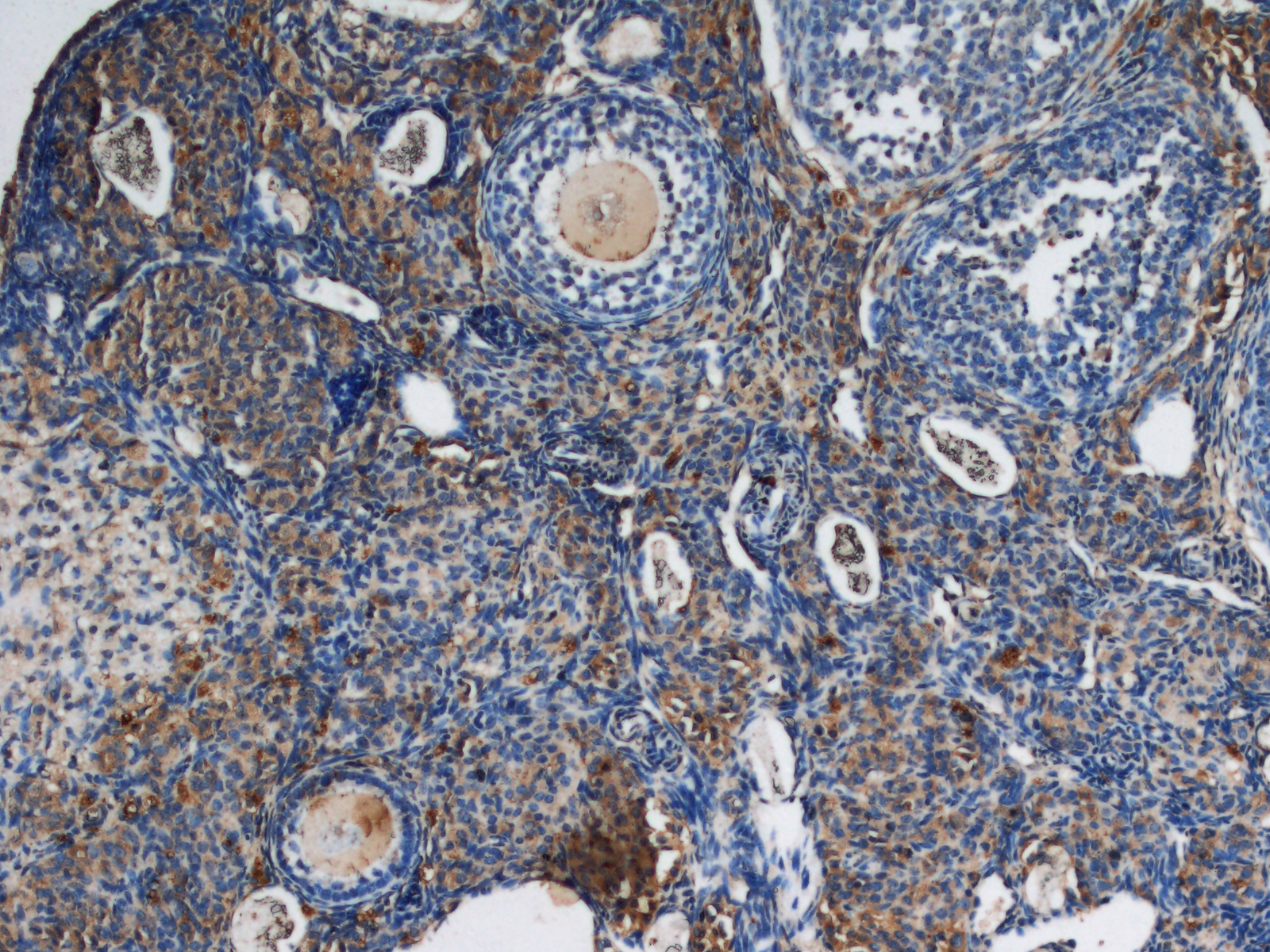

Supplement: S1 File — Significant increases in 4-HNE, NTY, and 8-OHdG immunostaining in ovarian interstitial cells and all follicle components were observed in the OI group. (ZIP) [file pone.0162194.s001.zip › S1 and S1 File. Immunohistochemistry pictures for each marker/4-HNE positive control.tif]

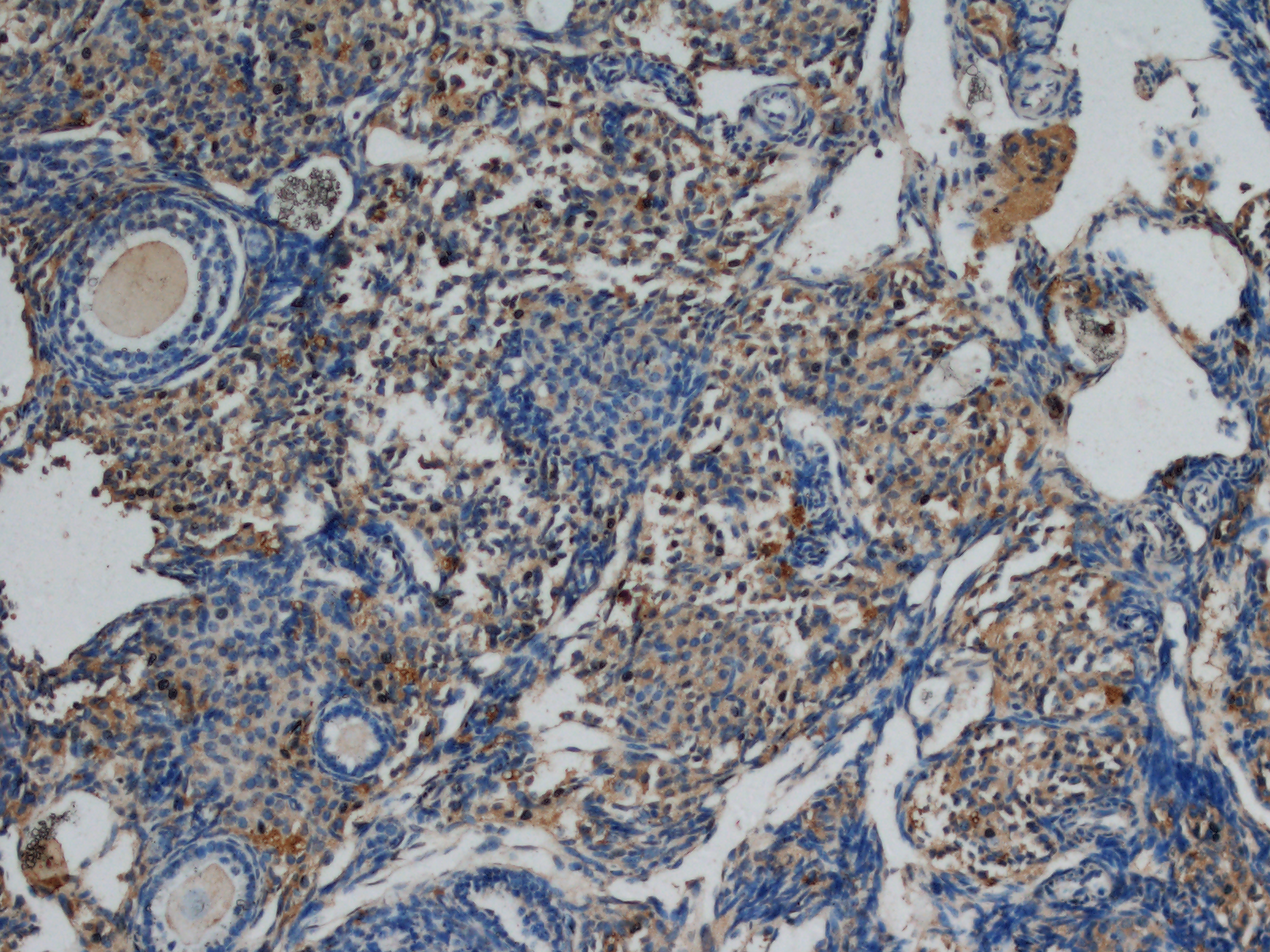

Supplement: S1 File — Significant increases in 4-HNE, NTY, and 8-OHdG immunostaining in ovarian interstitial cells and all follicle components were observed in the OI group. (ZIP) [file pone.0162194.s001.zip › S1 and S1 File. Immunohistochemistry pictures for each marker/8-OHdG positive control (2).tif]

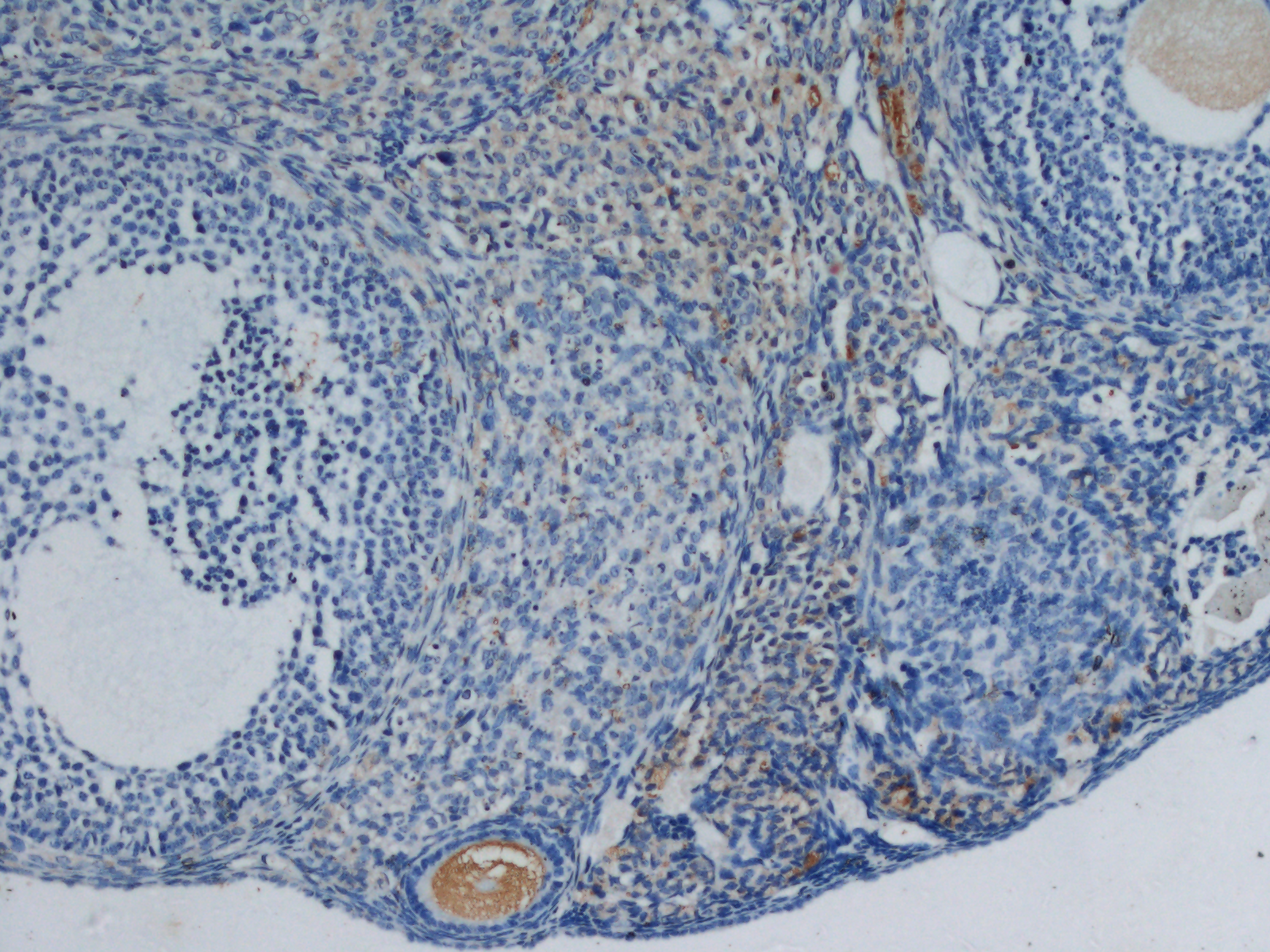

Supplement: S1 File — Significant increases in 4-HNE, NTY, and 8-OHdG immunostaining in ovarian interstitial cells and all follicle components were observed in the OI group. (ZIP) [file pone.0162194.s001.zip › S1 and S1 File. Immunohistochemistry pictures for each marker/NC 4-HNE (3).tif]

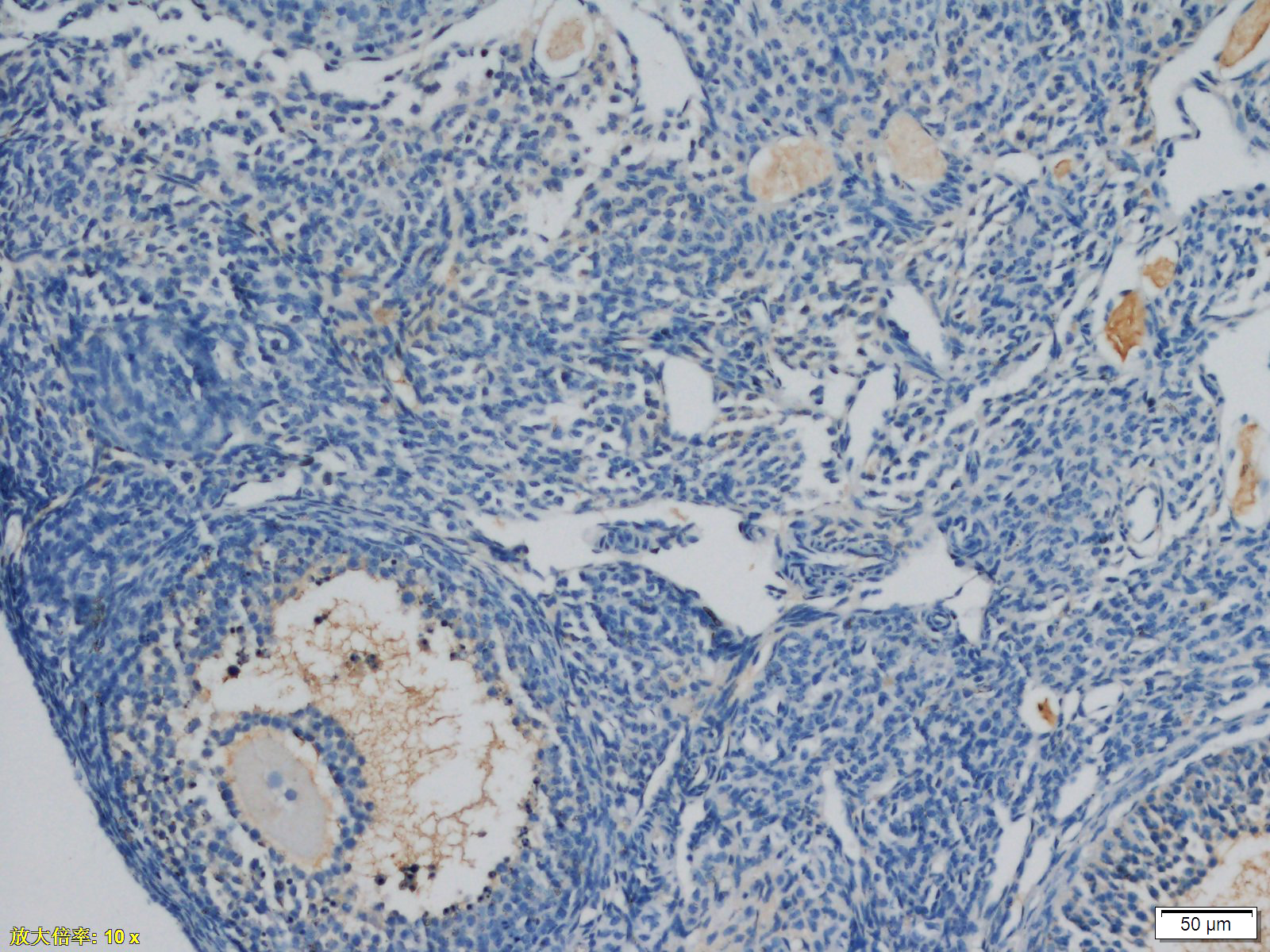

Supplement: S1 File — Significant increases in 4-HNE, NTY, and 8-OHdG immunostaining in ovarian interstitial cells and all follicle components were observed in the OI group. (ZIP) [file pone.0162194.s001.zip › S1 and S1 File. Immunohistochemistry pictures for each marker/NC 8-OHdG.tif]

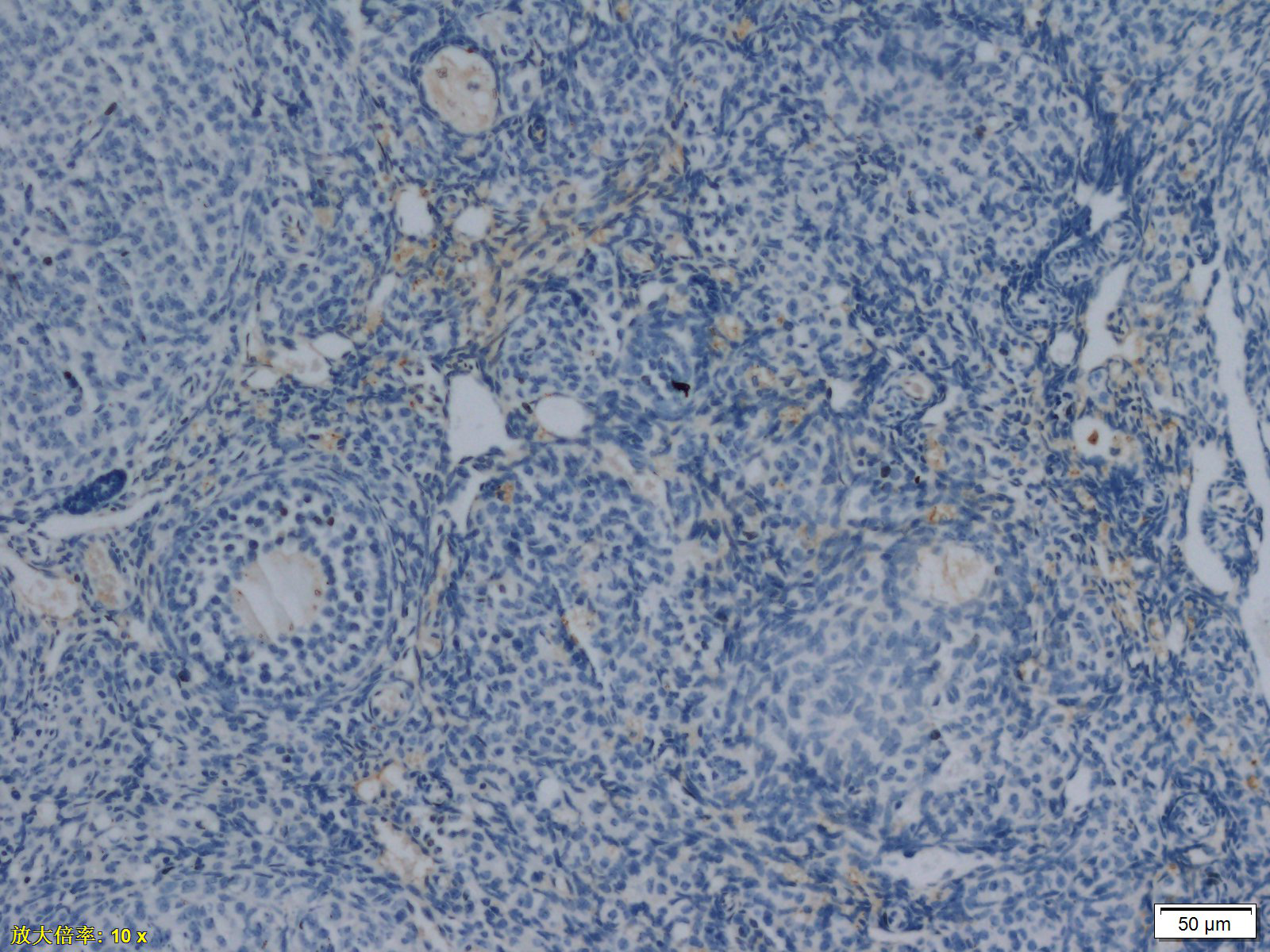

Supplement: S1 File — Significant increases in 4-HNE, NTY, and 8-OHdG immunostaining in ovarian interstitial cells and all follicle components were observed in the OI group. (ZIP) [file pone.0162194.s001.zip › S1 and S1 File. Immunohistochemistry pictures for each marker/NC NTY.tif]

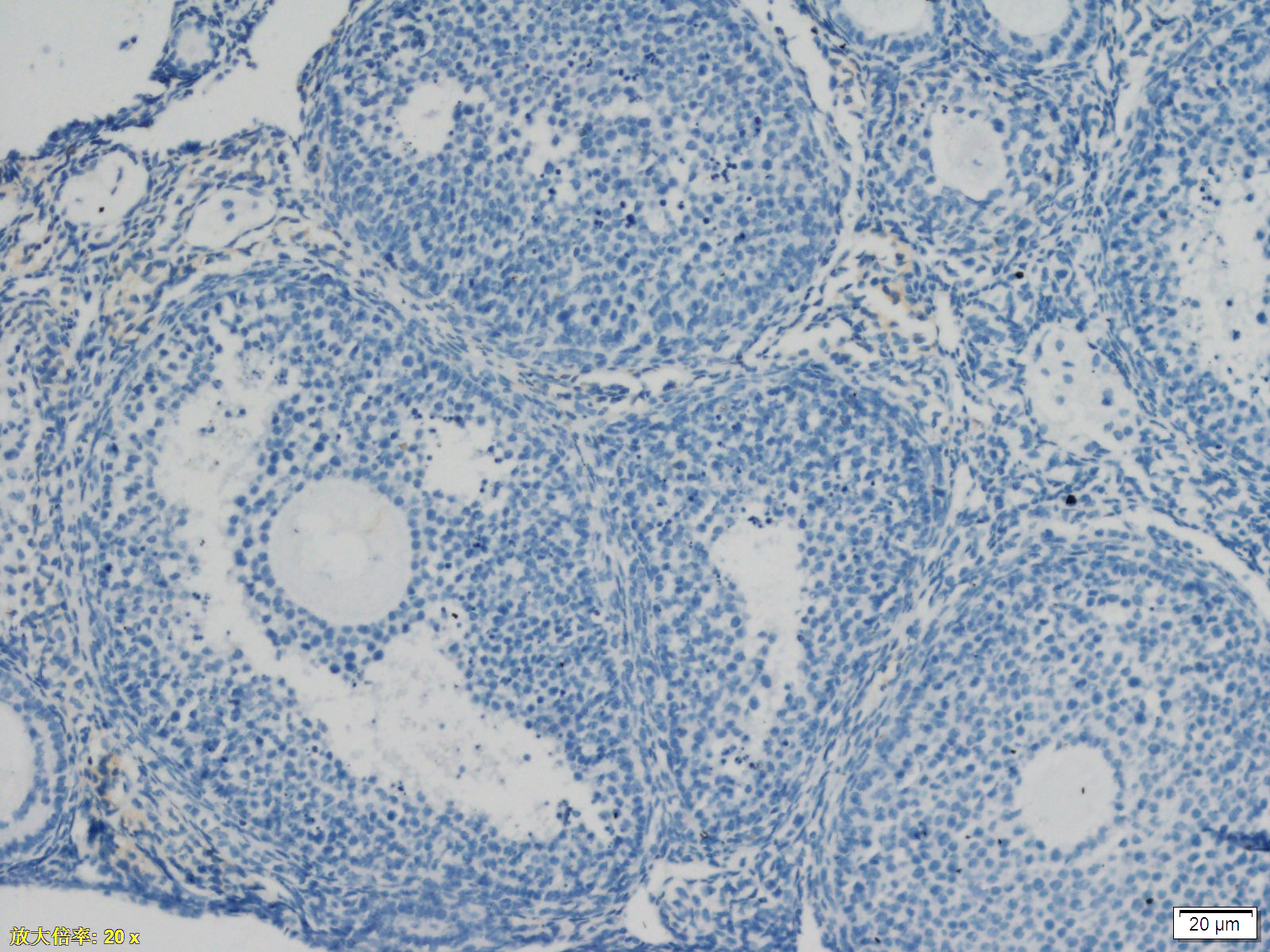

Supplement: S1 File — Significant increases in 4-HNE, NTY, and 8-OHdG immunostaining in ovarian interstitial cells and all follicle components were observed in the OI group. (ZIP) [file pone.0162194.s001.zip › S1 and S1 File. Immunohistochemistry pictures for each marker/Negative control 4-HNE .tif]

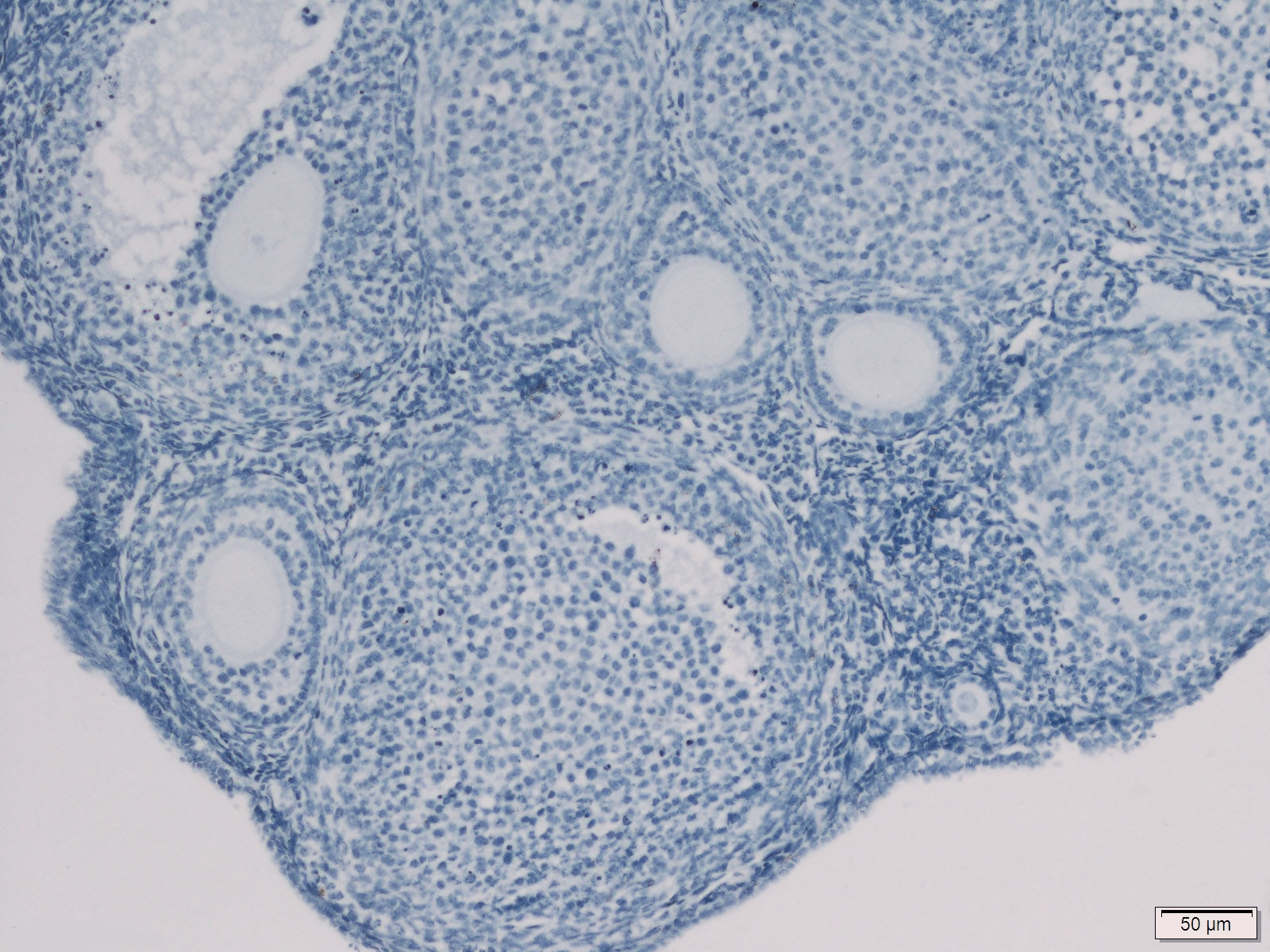

Supplement: S1 File — Significant increases in 4-HNE, NTY, and 8-OHdG immunostaining in ovarian interstitial cells and all follicle components were observed in the OI group. (ZIP) [file pone.0162194.s001.zip › S1 and S1 File. Immunohistochemistry pictures for each marker/Negative control NTY.tif]

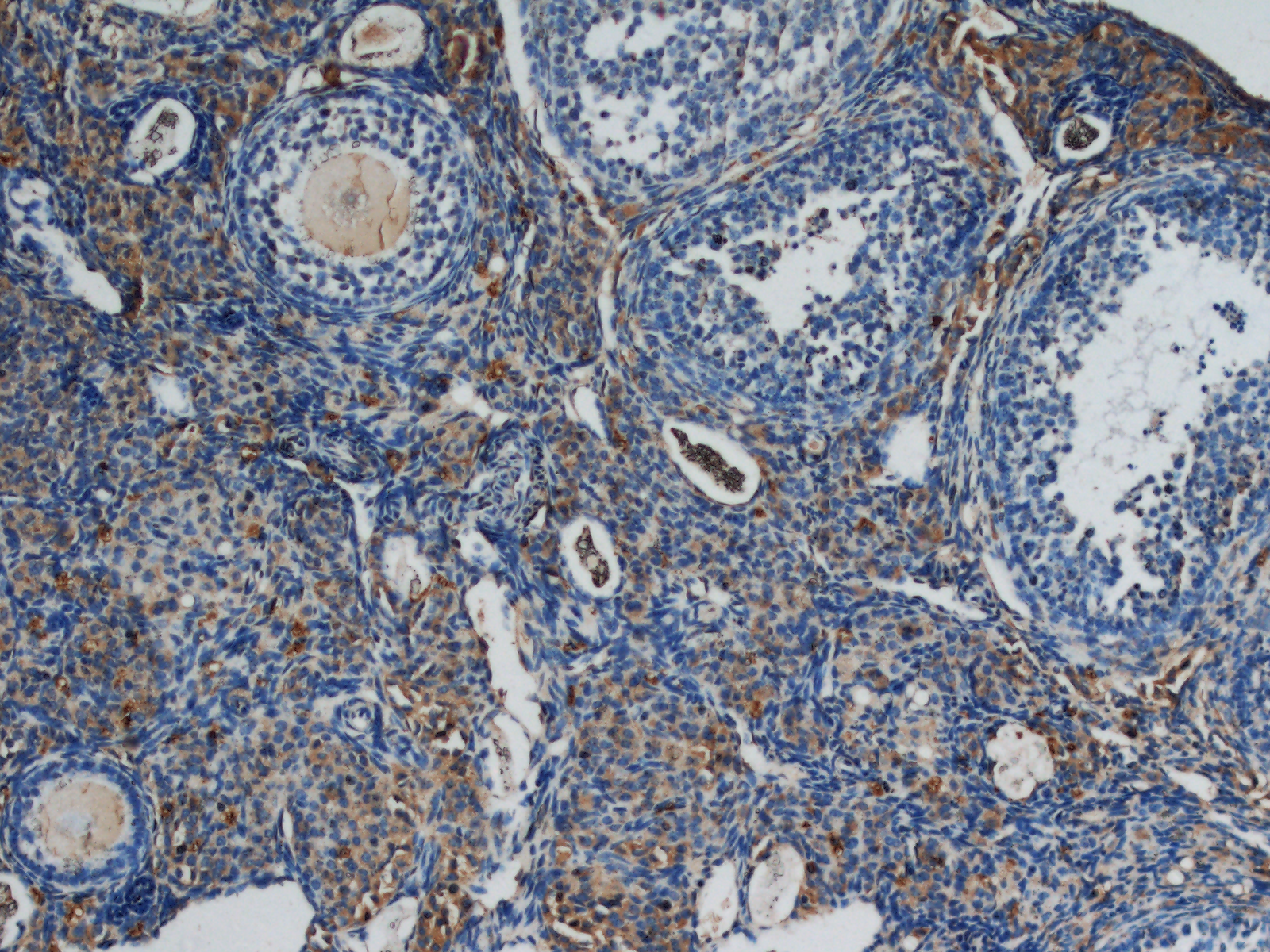

Supplement: S1 File — Significant increases in 4-HNE, NTY, and 8-OHdG immunostaining in ovarian interstitial cells and all follicle components were observed in the OI group. (ZIP) [file pone.0162194.s001.zip › S1 and S1 File. Immunohistochemistry pictures for each marker/OI 4-HNE.tif]

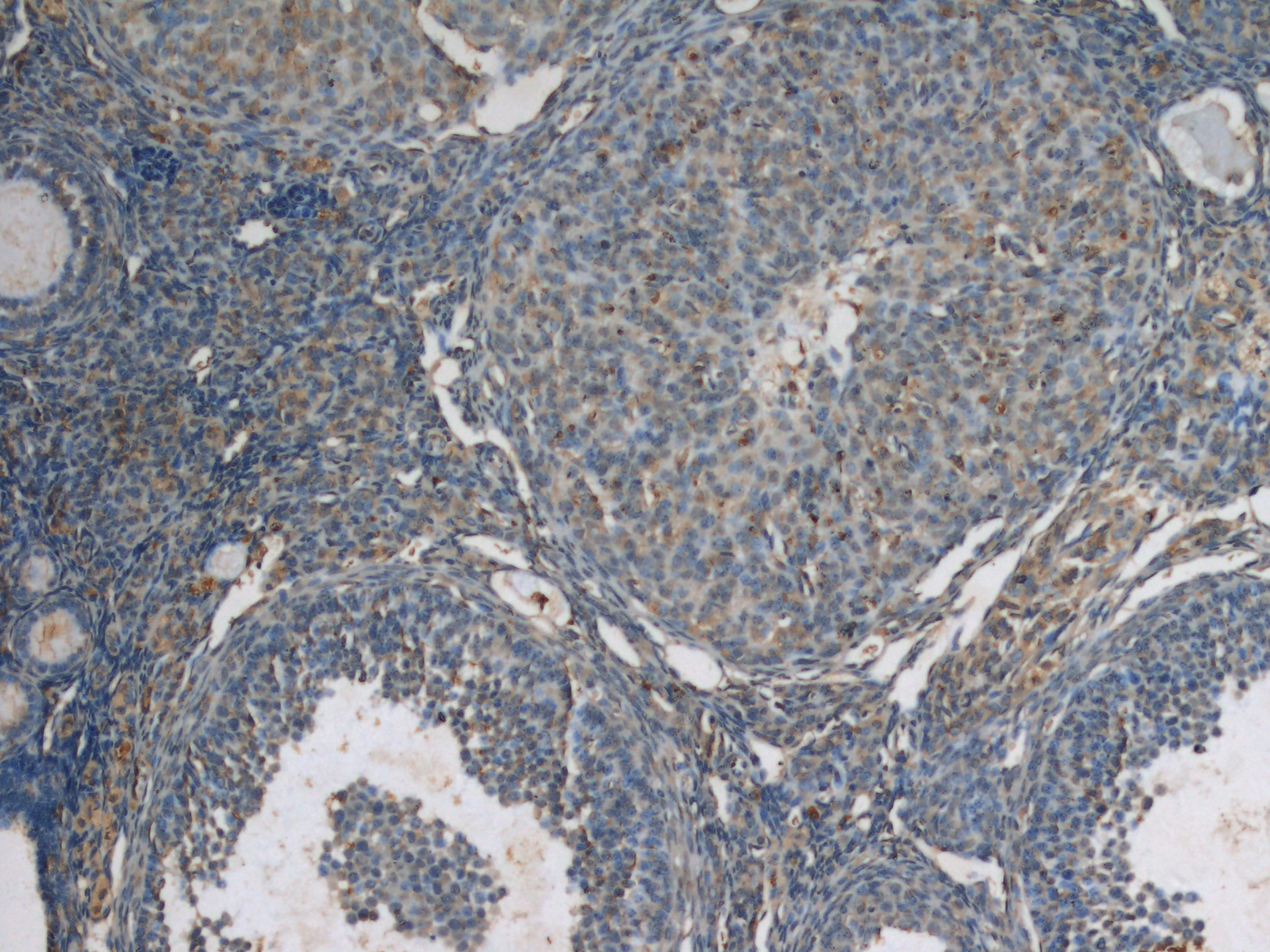

Supplement: S1 File — Significant increases in 4-HNE, NTY, and 8-OHdG immunostaining in ovarian interstitial cells and all follicle components were observed in the OI group. (ZIP) [file pone.0162194.s001.zip › S1 and S1 File. Immunohistochemistry pictures for each marker/OI 8-OHdG (2).tif]

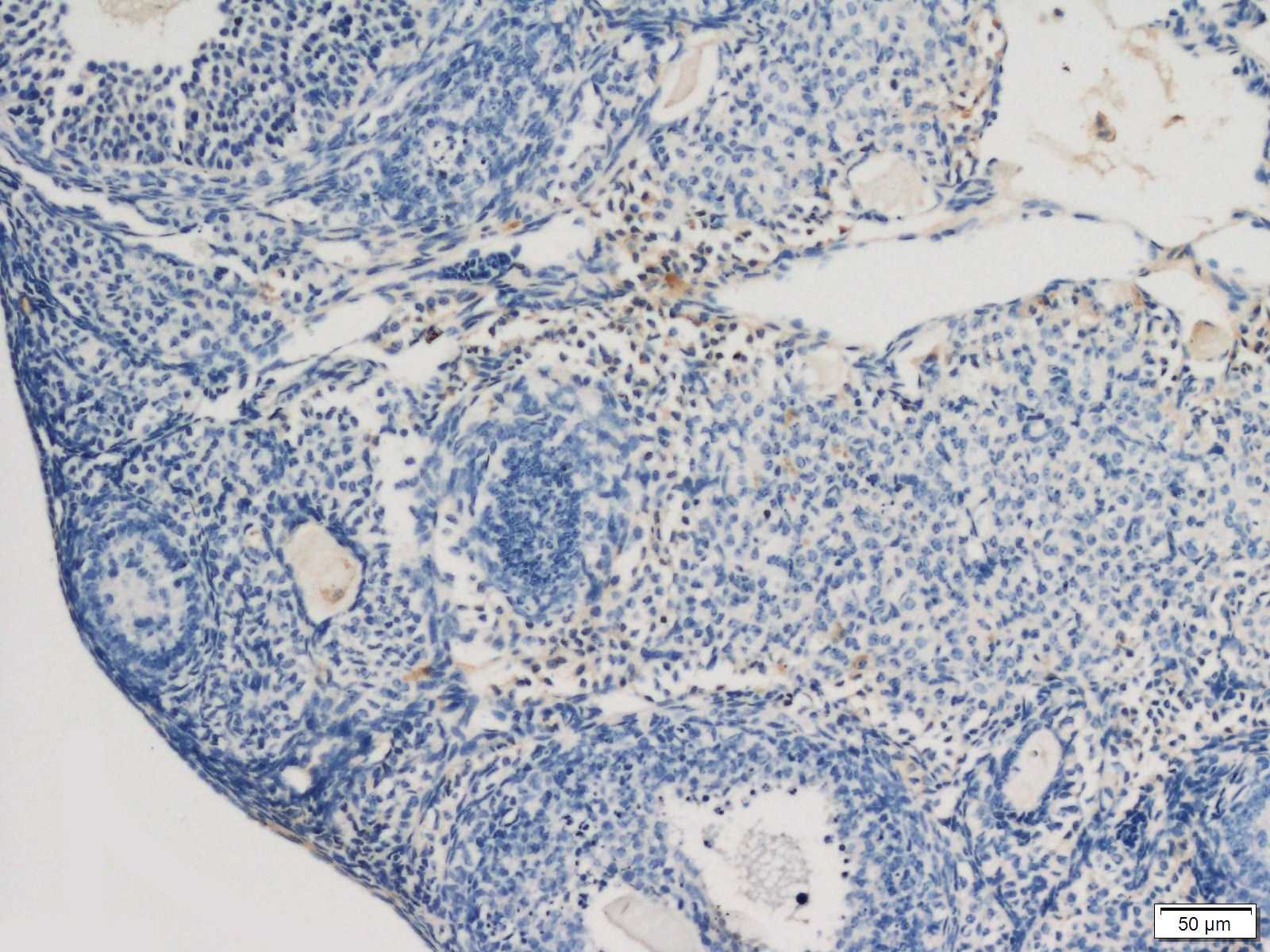

Supplement: S1 File — Significant increases in 4-HNE, NTY, and 8-OHdG immunostaining in ovarian interstitial cells and all follicle components were observed in the OI group. (ZIP) [file pone.0162194.s001.zip › S1 and S1 File. Immunohistochemistry pictures for each marker/OI NTY .tif]

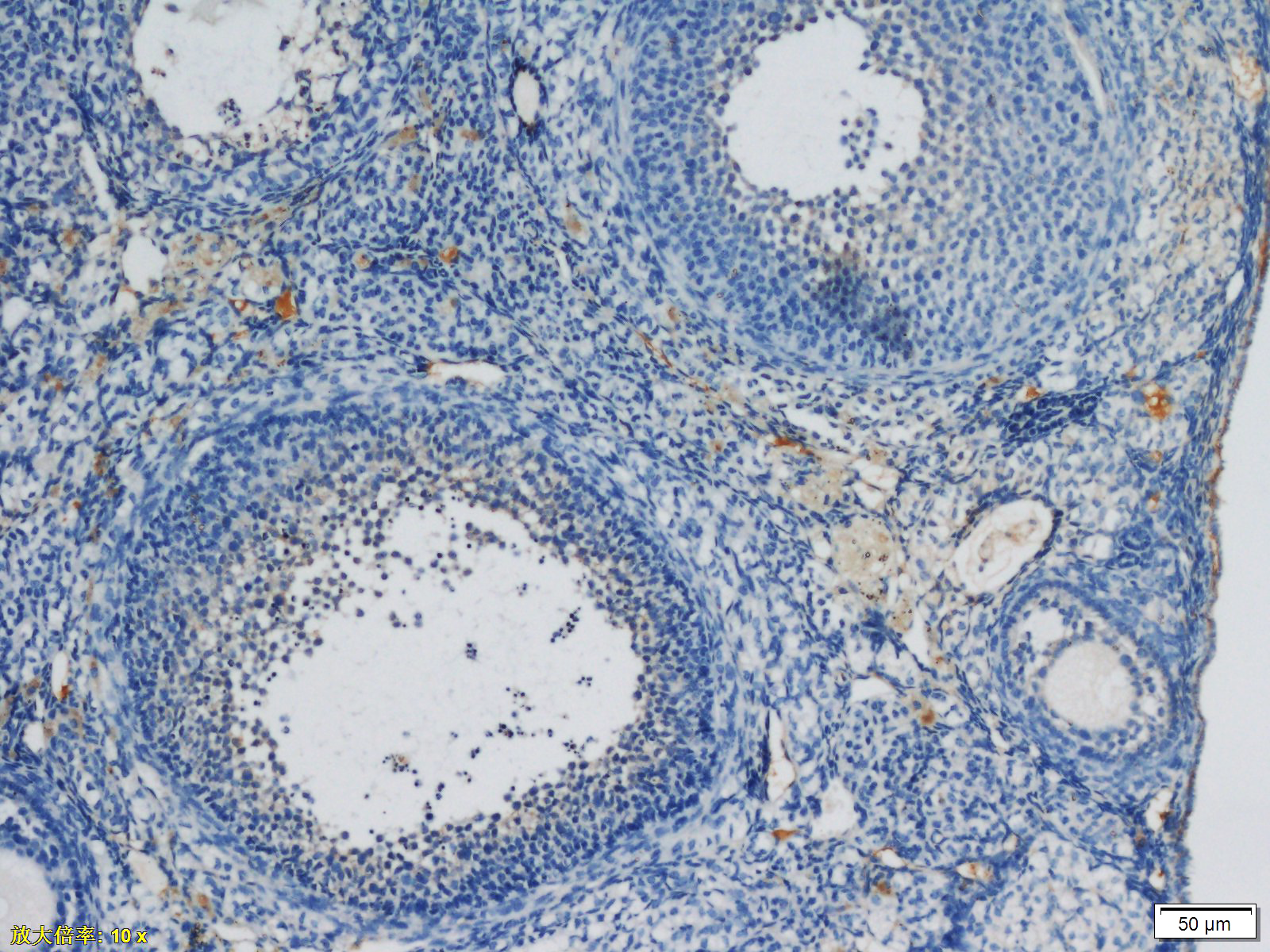

Supplement: S1 File — Significant increases in 4-HNE, NTY, and 8-OHdG immunostaining in ovarian interstitial cells and all follicle components were observed in the OI group. (ZIP) [file pone.0162194.s001.zip › S1 and S1 File. Immunohistochemistry pictures for each marker/Positive control NTY.tif]
